# The treatment practices for anterior urethral strictures in China: A case-based survey

**DOI:** 10.3389/fsurg.2022.863463

**Published:** 2022-07-27

**Authors:** Changhao Hou, Jiahao Lin, Yubo Gu, Wei Yuan, Zeyu Wang, Xianjie Xiu, Qiang Fu, Lujie Song

**Affiliations:** ^1^ Department of Urology, Shanghai Jiao Tong University Affiliated Sixth People’s Hospital, Shanghai, China; ^2^ Shanghai Eastern Institute of Urologic Reconstruction, Shanghai Jiao Tong University Affiliated Sixth People's Hospital, Shanghai, China

**Keywords:** anterior urethral strictures, treatment, urethroplasty, recommendation, case-based survey

## Abstract

**Objective:**

To investigate the treatment concept of Chinese urologists for anterior urethral strictures based on actual cases.

**Methods:**

A self-designed case-based questionnaire was distributed to the members of Official WeChat account of Learning Union from March 19, 2020, to April 10, 2020. Questionnaires requested respondents' demographic information and responses to five cases of anterior urethral stricture: short obliterative bulbar urethral stricture caused by straddle injury (Case 1), idiopathic bulbar urethral stricture after failure of multiple endoscopic therapy (Case 2), iatrogenic long penile urethral stricture (Case 3), lichen sclerosis-related urethral stricture (Case 4), and anterior urethral stricture in indwelling catheter after multiple failure of endoscopic surgery (Case 5). Data was described by frequency and percentage.

**Results:**

A total of 1,267 valid anonymous questionnaires were received. Urethroplasty was recommended more frequently than endoscopic surgery (Case 1: 47.8% vs. 32.8%,Case 2: 42.5% vs. 33.8%, Case 3: 36.1% vs. 26.7%). Referrals patients to other urologists engaged in urethral repair and reconstruction account for a high portion of the treatment (Case 1:18.4%, Case 2:23.1%, Case 3:36.5%, Case 4:27.7%,Case 5:9.3%). Excision and primary anastomosis urethroplasty (EPA) was preferred for treatment of Case 1 (42.5%). For Case 2, the most popular choice was EPA (30.6%). Although the patient has a history of failure in endoscopic surgery, 33.8% of urologists continue to choose endoscopic surgery. For Case 3, 20.0% of urologists would perform oral mucosal urethroplasty. Surprisingly, 5.9% chose EPA. For Case 4, 37.3% of urologists selected meatotomy, 30.4% suggested that glans and urethral biopsies should be performed. 21.0% chose to use steroid ointment after surgery. For Case 5, 26.3% of the respondents believed that urethrography should be performed after removing catheter more than one week, if the urine is obstructed during the period, performing cystostomy firstly.

**Conclusions:**

In China, the concept of urethroplasty is more widely accepted than endoscopic surgery for the treatment of anterior urethral strictures. The concept of referral has been widely formed among Chinese urologists. Better understanding of the comprehensive treatment of lichen sclerosis related anterior urethral stricture and the principle of urethral rest should be strengthened.

## Introduction

Anterior urethral strictures (AUS) are common refractory urological diseases (1), accounting for over 90% of urethral strictures in the developed world (2, 3). They cause various clinical symptoms, including voiding dysfunction, hematuria, urinary stream weakening, or pain, seriously diminishing the quality of life for patients. Meanwhile, AUS have very different characteristics depending on their location, length, etiology, and degree of fibrosis; it would be naive to apply the same procedures indiscriminately in all cases (4). The treatment of AUS has undergone significant changes over time, from minimally invasive surgery with different degrees of success, to definitive urethroplasty. However, there is no consensus regarding the treatment of this disease (5).

We designed this survey to better understand the clinical practices of Chinese urologists for treatment of this disease. In this study, given the diversity of types of AUS, we chose a case-based investigation method in an attempt to highlight areas of consistency and variability. This study surveyed the management of AUS and compared it with recent AUS guidelines and the treatment actually received by the patient, hoping to reflect the concept of management practices of AUS in China.

## Materials and methods

### Questionnaire

We designed an anonymous questionnaire and selected five representative cases of AUS (see Supplementary Material). Each of the five patients had a different subtype of AUS (long or short, traumatic or iatrogenic, bulbar or penile). All presented cases were surgically treated at our institution between 2019 and 2020. Patient information included age, sex, etiology, imaging examination, penile appearance, and alternative treatment options. Furthermore, this questionnaire also queried respondents about demographic information. Case 4 was a multiple-choice response, while the others required single choices.

We used the Official WeChat account of Learning Union to distribute the questionnaire. We set that each urologist can only response in once. All enrolled urologists signed a digital informed consent form before accessing the questionnaire online.

### Statistical analysis

Excel software was used for data collection, and the SPSS software package (version 20.0; SPSS Inc., Chicago, IL, USA) was used for statistical analysis. Baseline demographic characteristics and treatment of AUS were described by frequency and percentage.

## Results

### Cases

#### Case 1: Short obliterative bulbar urethral stricture caused by straddle injury

**Figure 1:**
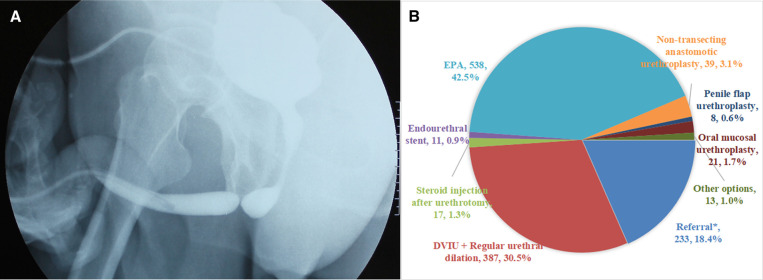
(**A**) The urethrography of Case 1. (**B**) Management recommendations of urologists for Case 1.

A 45-year-old male, unable to urinate normally because of the straddle injury 4 months ago. At present, urinary drainage depends on cystostomy (Figure 1A):

In fact, excision and primary anastomosis (EPA) urethroplasty was performed to Case 1. Judging from the results of the survey, nearly half the urologists (42.5%) would perform an EPA, followed by direct vision internal urethrotomy (DVIU) combined with regular urethral dilation (30.5%). 18.4% of urologists referred patients to other urologists engaged in urethral repair and reconstruction (Figure 1B). For this patient with obliterative bulbar urethral stricture caused by straddle injury, there are still 3.1% respondents who chose non-transecting anastomotic urethroplasty.

#### Case 2: Idiopathic bulbar urethral stricture after failure of multiple endoscopic surgery

**Figure 2:**
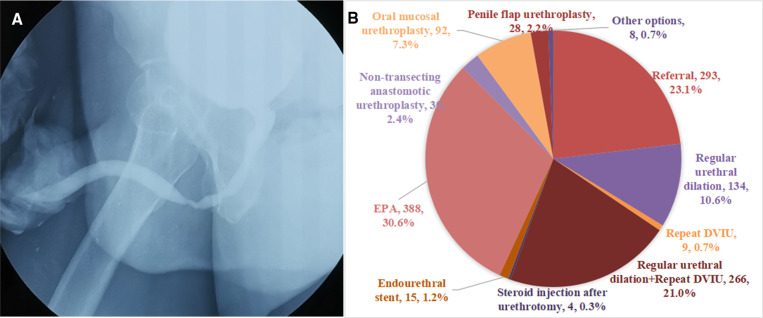
(**A**) The urethrography of Case 2. (**B**) Management recommendations of urologists for the Case 2.

A 36-year-old male, with unexplained dysuria for 3 years. Two DVIU were performed in the past year, and all of them recurred. Urethral dilation was not performed regularly. The urethrography revealed bulbar urethral stricture with a length of about 2 cm. The maximum urinary flow rate is 6ml/s (Figure 2A).

In fact, oral mucosal urethroplasty was performed to Case 2. Judging from the results of the survey, the most popular choice is EPA (30.6%). Although the patient has a history of failure in endoscopic surgery, some urologists continue to choose endoscopic surgery such as DVIU (21.7%). For this case of idiopathic bulbar urethral stricture, 7.3% of urologists chose oral mucosal urethroplasty and 2.4% chose non-transecting anastomotic urethroplasty (Figure 2B).

#### Case 3: Iatrogenic long penile urethral stricture

**Figure 3:**
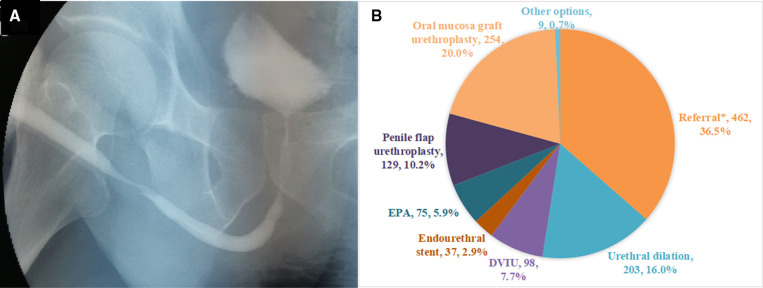
(**A**) The urethrography of Case 3. (**B**) Management recommendations of urologists for the Case 3.

A 44-year-old male, had a history of indwelling catheter because of traumatic brain injury half a year ago, with dysuria and weakening of the urinary stream. Currently, recovery from the traumatic brain injury was progressing well. Urethrography showed that the stricture was located in the penile urethra with a length of about 3.5 cm; The maximum urinary flow rate was 7 ml/s (Figure 3A).

In fact, Case 3 underwent oral mucosal urethroplasty. Judging from the results of the survey, more than one-third of urologists (36.5%) suggested referral to other urologists specializing in urethral repair and reconstruction. 20.0% of urologists would perform oral mucosal urethroplasty, which is higher than that of penile flap urethroplasty (10.2%). Surprisingly, 5.9% of urologists chose EPA (Figure 3B).

#### Case 4: Lichen sclerosis (LS)-related AUS

**Figure 4:**
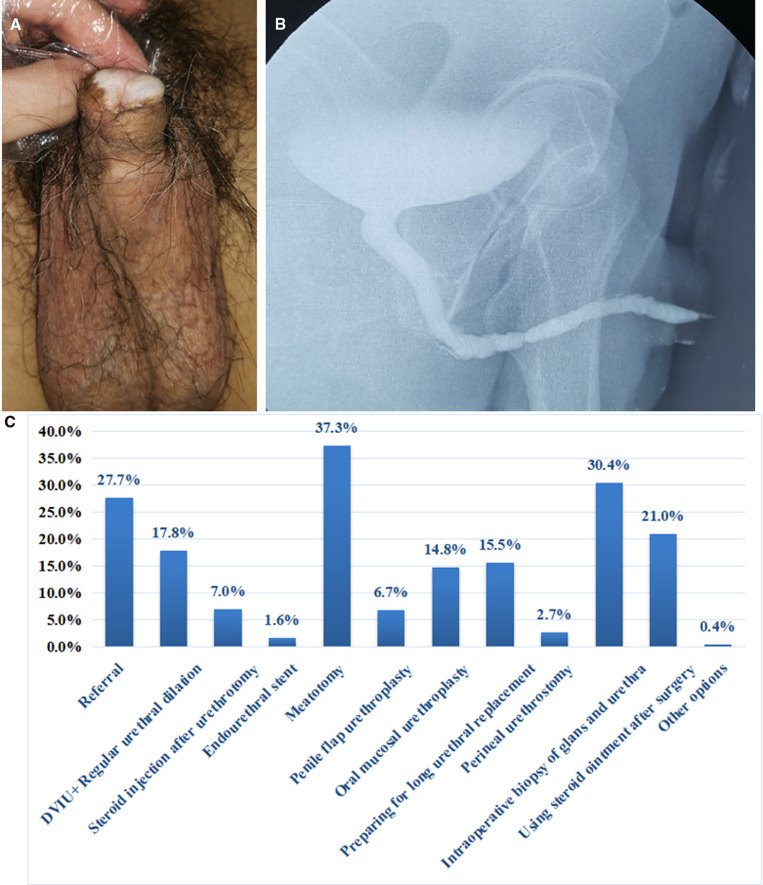
(**A**) Penile appearance of Case 4. (**B**) The urethrography of Case 4. (**C**) Management recommendations of urologists for the Case 4.

A 50-year-old male, underwent circumcision 30 years ago, and then gradually developed dysuria. Urethral dilatation was performed many times because of urethral stricture, which was effective in a short time and the urinary stream gradually decreased after dilatation. At present, the maximum urinary flow rate is 4 ml/s, and the residual urine is 30 ml. The appearance of penis is shown in Figure 4A and urethrography is shown in Figure 4B.

In fact, for Case 4, the one-stage augmentation anterior urethroplasty for the treatment of panurethral stricture. Glans and urethra biopsies were performed and steroid ointment was applied postoperatively, and regular follow-up was recommended to the patient. Judging from the results of the survey, the most common choice is meatotomy (37.3%). The proportion of oral mucosa urethroplasty is more than penile flap urethroplasty (14.8% vs. 6.7%). Despite a history of multiple failed urethral dilation, 17.8% of urologists continue to choose urethral dilation. 30.4% suggested that glans and urethral biopsies should be performed. 21.0% of urologists would treat the patient with steroid ointment after surgery.

#### Case 5: AUS in indwelling catheter after multiple failure of endoscopic surgery

A 66-year-old male presented with dysuria after transurethral resection of the prostate (TURP) for more than 2 years. Anterior urethral dilation was performed for many times in the other hospital, which was effective for a short time, and the urinary stream gradually decreased after dilatation. Due to acute urinary retention 3 days ago, 20 French Foley catheter was indwelled after urethral dilation. At present, the catheter is in indwelling and the drainage is unobstructed.

**Figure 5:**
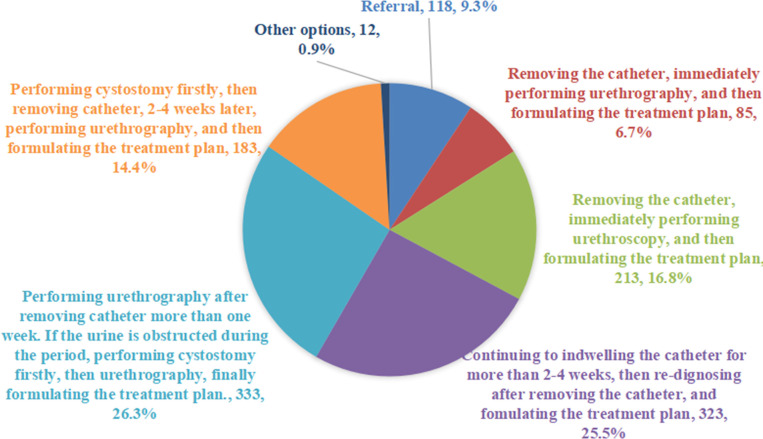
Management recommendations of urologists for the Case 5.

In fact, for Case 5, we believed that urethrography should be performed after removing catheter more than one week, if the urine is obstructed during the period, performing cystostomy firstly, then urethrography, finally formulating the treatment plan. Judging from the results of the survey, 26.3% of the respondents agreed with our choice. 25.5% of respondents chose to continue to indwelling catheter for more than 2-4 weeks, then re-dignosing after removing catheter, and formulating the treatment plan. 14.4% of urologists thought that the cystostomy should be performed firstly, then removing the catheter, 2–4 weeks later, performing urethrography, finally formulating the treatment plan. In addition, 6.7% and 16.8% of the urologists thought that the catheter should be removed firstly and urethrography or urethroscopy should be performed immediately (Figure 5).

## Discussion

To the best of our knowledge, this is the first case-based survey of AUS. In the previous survey for the diagnosis and treatment of AUS based on urologists' clinical experience, we found that the treatment of AUS in China is still dominated by endoscopic surgery, with most urologists using the reconstructive ladder treatment strategy (6). However, this survey is divorced from clinical practice, mainly to understand the respondents' clinical experience, rather than the treatment concept of AUS. Therefore, we further conducted the survey based on specific cases. It can effectively reflect the differences between cases, reproduce the disease scenario for the process of diagnosis and treatment.

Case 1–5 presented with short obliterative bulbar urethral stricture caused by straddle injury, idiopathic bulbar urethral stricture after failure of multiple endoscopic surgery, iatrogenic long penile urethral stricture, LS-related AUS, and AUS in indwelling catheter after multiple failure of endoscopic surgery, respectively. Due to differences such as those in location, length, and medical history, the treatment of AUS is diverse. Considering the wide variety of AUS cases, we chose a case-based approach to closely represent real clinical situations.

### Urethroplasty is more popular than endoscopic surgery

Overall, more urologists chose urethroplasty than endoscopic surgery in Case 1 to 3 (Case 1: 47.8% vs. 32.8%,Case 2: 42.5% vs. 33.8%, Case 3: 36.1% vs. 26.7%). This is different from our previous survey based on urologists' experience (6), mainly because the case-based survey can more truly reflect the clinical situation. For the Case 1, the urethral scar caused by straddle injury was usually severe, urethrography showed urethral obliteration, and the obliterative urethral segment was shorter. 42.5% of urologists would perform EPA, which allows for complete removal of scarred tissue and the restoration of urethral patency (7). The proportion of DVIU and urethral dilation is as high as 30.5%, which is not appropriate because the bulbar urethral scars caused by straddle injuries are often thick. Although endoscopic surgery can quickly relieve the symptoms of patients, it cannot completely remove the scar and has a high recurrence rate.

Many studies have shown that patients with a history of failure of endoscopic surgery have a high recurrence rate and increased complexity of urethral stricture (8). However, in Case 2, 32.3% of urologists still chose to perform urethral dilation or repeated DVIU. The American Urological Association (AUA) and European Urology Association (EAU) guidelines state that after endoscopic treatment fails, a repeat of endoscopic surgery is not recommended again, and should offer urethroplasty (1, 9). In addition, for this idiopathic bulbar urethral stricture, most urologists chose EPA (30.6%), which may be due to its simplicity and ease of learning compared with augmentation urethroplasty. Only 7.3% chose oral mucosa urethroplasty, 2.4% chose non-transecting anastomotic urethroplasty. In fact, this patient underwent oral mucosal urethroplasty and was successful. It also reflects that there are still differences in clinical practice and guidelines among Chinese urologists.

For penile urethral stricture (Case 3), AUA guidelines suggest that endoscopic treatment is less likely to succeed, requiring tissue transfer or staging treatment, and is not suitable for EPA (1). Surprisingly, 5.9% of urologists chose EPA. Our survey results shown that substitution urethroplasty is performed more frequently than EPA (30.2% vs. 5.9%). In the choice of substitutes, the AUA and EAU guidelines state that both penile flap and oral mucosa can be used, with no statistical difference in therapeutic effect. However, the EAU guidelines suggest that the penile flap is associated with increased morbidity and longer operation time (9). Our survey results shown that urologists prefer oral mucosa, which is also consistent with other studies (10) and the actual treatment.

### The understanding of AUS related to LS is still insufficient

Stricture related to LS is recognized as one of the refractory diseases. AUA guidelines state that management goals of LS should be to alleviate symptoms, prevent and treat urethral stricture, and prevent and detect malignant transformation (11). Therefore, the treatment of LS should be comprehensive. 37.3% of urologists selected meatotomy. As a simple treatment, meatotomy can relieve voiding obstruction to some extent. But it will affect the appearance of penis, which is the suboptimum treatment method. Although the patient had a history of repeated urethral dilation failures, 17.8% continued to chose DVIU and regular urethral dilation. 37.0% chose one-stage urethroplasty, and the proportion of oral mucosa was higher than that of penile flap (14.8% vs. 6.7%). There is some evidence that extended meatotomy in conjunction with high-dose topical steroids may decrease the risk of recurrence compared to that of meatotomy alone (12). 7.0% of urologists chose to use it. In addition, it is estimated that the incidence of penile cancer in patients with LS is between 2.3% and 8.4% (13, 14). The EAU guidelines list LS as an important risk factor for penile cancer (15). Biopsies can confirm the LS’s diagnosis and exclude malignant or premalignant changes. 30.4% of the respondents suggested a biopsy, and most urologists did not realize the value of biopsies and the use of steroid ointment. The results of previous studies in our institution shown that strictures related to LS were gradually recognized (16). However, the results of this survey shown the necessity of strengthening the understanding of LS and continuation of monitoring for penile cancer. Of course, some urologists may not recognize LS’s penile appearance or panurethral stricture revealed by urethrography. This may be the reason for the great difference in the results.

### Urethral rest should be paid attention to

For Case 5, the patient has been indwelling catheter for 72 h. 25.5% of respondents chose to continue to indwell catheter for more than 2–4 weeks. This is different from the AUA guidelines, which suggest that catheters may be safely removed after 24–72 h following uncomplicated dilation or DVIU (1).

The most common choice is that urethrography should be performed after removing catheter more than one week, if the urine is obstructed during the period, performing cystostomy firstly. However, a total of 23.5% chose to remove catheters and formulate treatment strategies after re-evaluation by cystoscopy or urethrography immediately, which is inappropriate. Here, we introduce the concept of urethral rest. Urethral rest has been defined as freedom from urethral instrumentation for a period of time or suprapubic cystostomy (SPC) tube placement (17). The AUA and EAU guidelines said that a period of urethral rest is necessary after any form of urethral manipulation (1, 9). Ojima et al. (18) believed that the appropriate time of urethral rest promoted the identification of severely fibrotic stricture segments, which is consistent with our clinical practice.

### Referral to a professional urologist for urinary repair and reconstruction is an important recommendation

Surprisingly, the referral rate remained high in all types of AUS (Case 1:18.4%, Case 2:23.1%, Case 3:36.5%, Case 4:27.7%,Case 5:9.3%). This shown that the management of AUS remains a challenge. In view of the important role of referrals in AUS management, researchers have focused on pre-referral treatment. Ojima et al. (18) retrospectively analyzed the pre-referral treatment medical records of 371 patients with AUS. The results shown that transurethral procedures are often inappropriately used for pre-referral, and repeat transurethral procedures are considered inappropriate in any circumstances (18). These findings are consistent with the expert recommendations in the AUA guidelines (1). To the best of our knowledge, a referral model has not yet been firmly established, but according to our survey results, the concept of referral has been formed among some Chinese urologists.

Our study has some limitations that must be considered when interpreting the results. Firstly, our survey only selected five cases, which could not represent all the clinical scenarios of AUS. However, these cases represent the main clinical challenges of AUS and are representative. Secondly, from the perspective of geographical distribution, this study provides a widely representative sample of national practice. However, a selection bias toward urologists interested in urethral repair may be indicated, which limits the universality of the results. An online questionnaire may deter elderly urologists who do not use the internet regularly. Thirdly, although these cases are real, respondents did not observe or examine the patients, which may influence the formulation of their treatment strategies.

In conclusion, this presented case-based survey shown that in some respects the respondents' choices followed the guidelines for the diagnosis and treatment of AUS. The concept of urethroplasty is more widely accepted than endoscopic surgery for the treatment of AUS. Referral patients to the urologists engaged in urethral repair and reconstruction is an important solution in the treatment of complex AUS. Better understanding of the comprehensive treatment of LS-related AUS and the principle of urethral rest should be strengthened. Therefore, the promotion of appropriate strategies for AUS treatment is necessary among general urologists.

## Data Availability

The original contributions presented in the study are included in the article/**Suplementary Material**, further inquiries can be directed to the corresponding author/s.
