# Supplementary material for: The treatment practices for anterior urethral strictures in China: A case-based survey
Source: Front Surg. 2022 Jul 27;9:863463. doi: 10.3389/fsurg.2022.863463 (PMC9363623; doi:10.3389/fsurg.2022.863463)
Supplement: Supplementary file 1 [file Data_Sheet_1_v1.docx]

Supplementary Material

**Questionnaire on the treatment practices for anterior urethral strictures in China: a case-based survey**

**1. Case 1：**

A 45-year-old male, unable to urinate normally because of the straddle injury 4 months ago. At present, urinary drainage depends on cystostomy (Figure 1 A):

**Supplementary Figure 1：**The urethrography of Case 1

• Referrals to other urologists engaged in urethral repair and reconstruction.

• Direct vision internal urethrotomy (DVIU) + Regular urethral dilation

• Steroid injection after urethrotomy

• Endourethral stent

• Excision and primary anastomosis urethroplasty (EPA)

• Non-transecting anastomotic urethroplasty

• Penile flap urethroplasty

• Oral mucosal urethroplasty

• Other (Please describe):___________

**2. Case 2：**

A 36-year-old male, with unexplained dysuria for 3 years. Two DVIU were performed in the past year, and all of them recurred. Urethral dilation was not performed regularly. The urethrography revealed bulbar urethral stricture with a length of about 2 cm. The maximum urinary flow rate is 6ml/s (Figure 2 A).


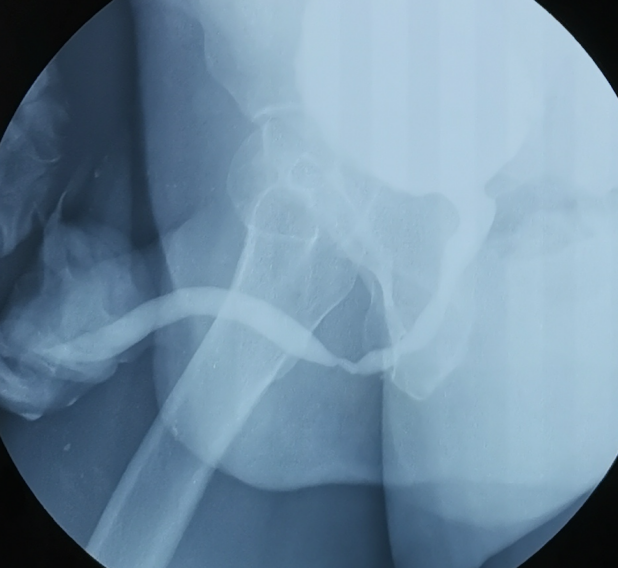


**Supplementary Figure 2：**The urethrography of Case 2

• Referrals to other urologists engaged in urethral repair and reconstruction.

• Regular urethral dilation

• Repeat DVIU

• Regular urethral dilation and repeat DVIU

• Steroid injection after urethrotomy

• Endourethral stent

• EPA

•Non-transecting anastomotic urethroplasty

• Oral mucosal urethroplasty

• Penile flap urethroplasty

• Other (Please describe):___________

**3. Case 3：**

A 44-year-old male, had a history of indwelling catheter because of traumatic brain injury half a year ago, with dysuria and weakening of the urinary stream. Currently, recovery from the traumatic brain injury was progressing well. Urethrography showed that the stricture was located in the penile urethra with a length of about 3.5 cm; The maximum urinary flow rate was 7 ml/s (Figure 3 A).


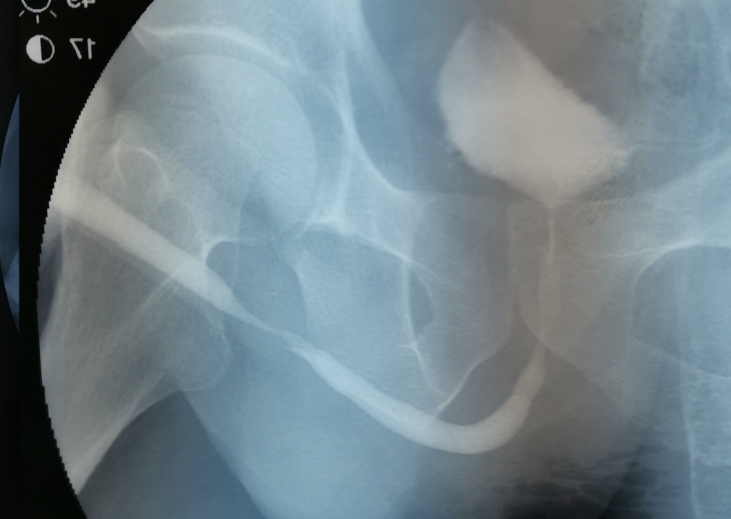


**Supplementary Figure 3：**The urethrography of Case 3

• Referrals to other urologists engaged in urethral repair and reconstruction.

• Urethral dilation

• DVIU

• Endourethral stent

• EPA

• Penile flap urethroplasty

• Oral mucosal urethroplasty

• Other (Please describe):__________

**4. Case 4：**

A 50-year-old male, underwent circumcision 30 years ago, and then gradually developed dysuria. Urethral dilatation was performed many times because of urethral stricture, which was effective in a short time and the urinary stream gradually decreased after dilatation. At present, the maximum urinary flow rate is 4ml/s, and the residual urine is 30 ml. The appearance of penis is shown in Figure 4 A and urethrography is shown in Figure 4 B.


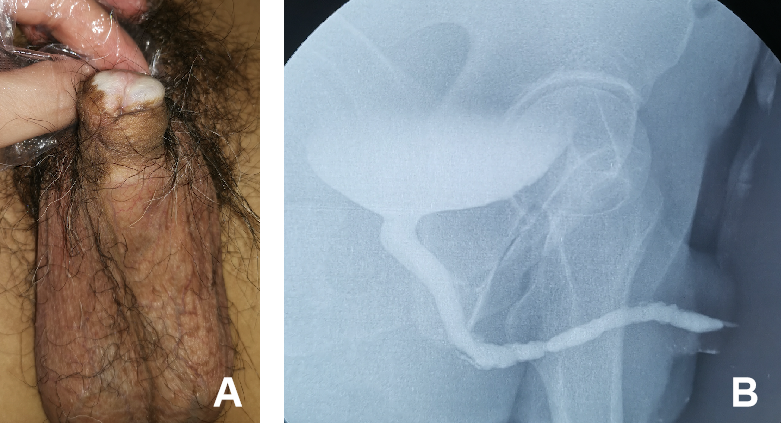


**Supplementary Figure 4.** The appearance of penis (A) and urethrography (B) of Case 4

• Referrals to other urologists engaged in urethral repair and reconstruction.

• DVIU+ Regular urethral dilation

• Steroid injection after DVIU

• Endourethral stent

• Meatotomy

• Penile flap urethroplasty

• Oral mucosal urethroplasty

• Preparing for long urethral replacement

• Perineal urethrostomy

• Intraoperative biopsy of glans and urethra

• Using steroid ointment after surgery

• Other (Please describe):___________

**5. Case 5：**

A 66-year-old male presented with dysuria after prostatectomy for more than 2 years. Anterior urethral dilation was performed for many times in the other hospital, which was effective for a short time, and the urinary stream gradually decreased after dilatation. Due to acute urinary retention 3 days ago, 20 French Foley catheter was indwelled after urethral dilation. At present, the catheter is in indwelling and the drainage is unobstructed.

• Referrals to other urologists engaged in urethral repair and reconstruction.

• Removing catheter, immediately performing urethrography, and then formulating a follow-up treatment plan

• Removing catheter, immediately performing urethroscopy, and then formulating a follow-up treatment plan

• Continuing to indwelling catheter for more than 2-4 weeks, then re-dignosing after removing catheter, and formulating a follow-up treatment plan

•Performing urethrography after removing catheter more than one week. If the urine is obstructed during the period, performing cystostomy firstly, then urethrography, finally formulating the treatment plan.

•Removing catheter, if urine is obstructed during the period, performing cystostomy firstly, then urethrography, finally formulating the treatment plan.

•Performing cystostomy first, then removing catheter, 2-4 weeks later, performing urethrography, and then formulating the follow-up treatment plan

• Other (Please describe):___________
